# From Invisibility to Inclusion: Evidence, Lived Experience, and Policy Directions for Multiple Chemical Sensitivity Report from the Resilience 2025 International Conference

**DOI:** 10.3390/ijerph23030280

**Published:** 2026-02-25

**Authors:** Karthik Vangala, John Molot, Adrianna Trifunovski, Rohini Peris

**Affiliations:** 1 Association pour la santé environnementale du Québec-Environmental Health Association of Québec (ASEQ-EHAQ), Saint-Sauveur, QC J0R 1R1, Canada; karthik@aseq-ehaq.ca (K.V.); johnmolot@aseq-ehaq.ca (J.M.); adrianna@aseq-ehaq.ca (A.T.); 2 Faculty of Medicine, University of Ottawa, Ottawa, ON K1H 8M5, Canada

**Keywords:** Multiple Chemical Sensitivity (MCS), scent-free policies, conference, Canada, Quebec, EHAC-ASEC, ASEQ-EHAQ, VOCs

## Abstract

**Highlights:**

**Public health relevance—How does this work relate to a public health issue?**

- The conference report highlights a groundbreaking 2-day event focusing on Multiple Chemical Sensitivity (MCS), a recognized disability which affects over a million Canadians (Canadian Community Health Survey, 2020), and millions more around the world, but it does not garner enough attention from the scientific community or policymakers.
- The report also highlights the underrecognized effects of fragrances and presents unique research about toxins in ubiquitous products and the benefits of fragrance-free policies.

**Public health significance—Why is this work of significance to public health?**

- MCS is a public health crisis that leads to disabling symptoms upon exposure to certain volatile organic compounds (VOCs) and other chemicals, affecting access to fundamental resources such as healthcare, dental care, housing, employment, and education, in addition to a loss of social belonging and quality of life.
- This report is an amalgamation of the knowledge presented during the conference, educating the public about the legitimate causes, effects, symptoms, and treatments of MCS, while presenting policies which promote inclusion and access for those affected.

**Public health implications—What are the key implications or messages for practitioners, policy makers and/or researchers in public health?**

- Researchers and public health institutions should allocate more resources to prioritize evidence-based MCS-focused research rooted in physiological effects and knowledge translation to address gaps in the evidence, reduce stigma, and support inclusive public health policies that protect vulnerable populations.
- Public health practitioners and policymakers must recognize fragrance and chemical exposures as avoidable environmental health risks and implement preventive measures such as scent-free policies, lowest-VOC-emission and least-toxic-product policies, advocate for product content transparency and indoor air quality guidelines that depend on appropriate product choices.

**Abstract:**

Organized by the Environmental Health Association of Canada (EHAC-ASEC) and the Environmental Health Association of Quebec (ASEQ-EHAQ), Resilience: An International Conference on Multiple Chemical Sensitivity, a groundbreaking event, was held online over two days, on 1 and 2 May 2025, and brought together experts and stakeholders from multiple sectors to address a condition that has received limited attention in health, policy and research contexts. The conference convened leading international researchers, legal experts, medical professionals, policymakers, and advocates to examine one of the most pressing but under-represented public health and human rights challenges of our time. Multiple Chemical Sensitivity (MCS) is a disabling condition affecting a growing number of individuals globally. Despite its prevalence, MCS remains marginalized and under-recognized in healthcare systems, policy frameworks, and scientific discourse. The conference spanned 2 days, drew nearly 900 participants, and featured engaging presentations and discussions that integrated scientific evidence, lived experience, and cross-sector perspectives. A central theme emerged: recognition of MCS extends beyond medical considerations, encompassing accessibility, gender equity, environmental justice and human rights.

## 1. Introduction

The Resilience: International Conference on Multiple Chemical Sensitivity (MCS), organized by the Environmental Health Association of Canada (EHAC-ASEC) and the Environmental Health Association of Quebec (ASEQ-EHAQ), was a landmark international event. It represented the first coordinated effort to unite the global MCS community, providing visibility and a platform for individuals who have long faced marginalization. A stated aim of the conference was to highlight emerging scientific evidence supporting the biological underpinnings of MCS and to encourage policy dialogue. The conference represented a significant effort to advance discussion and scientific exchange regarding this often-overlooked disability, with demonstrably positive results. This report provides a narrative summary of the scientific, clinical, legal, lived-experience, and policy discussions presented during the conference.

Multiple Chemical Sensitivity (MCS) is a chronic medical condition, the onset of which is often associated with repeated exposure to ubiquitous chemicals and pollutants. Symptoms are triggered by exposures to incitants, particularly volatile organic compounds (VOCs). These substances are commonly found in personal care products, laundry and cleaning agents, renovation materials, new furnishings, household products, and fragrances, as well as in other indoor pollutants and particulate matter [1]. Repeated exposure to these stressors can lead to sensitization and intolerance, with individuals reacting to chemical levels well below conventional thresholds, resulting in debilitating symptoms. These symptoms affect multiple body systems, including the dermal, respiratory, nervous, gastrointestinal, and cardiovascular systems [2]. Although symptom presentation varies between individuals, commonly reported symptoms include brain fog, runny nose, shortness of breath, headache, burning eyes, and fatigue [3].

MCS has emerged as a pressing public health concern. Data from the Canadian Community Health Survey (CCHS, 2020) indicate that nearly 4% of the population (approximately 1.3 million Canadians) report a diagnosis of MCS. Given increasing industrialization and widespread chemical exposures, several speakers noted concerns that prevalence may continue to rise, although further longitudinal data are needed. This trend can be observed in Canada, where the prevalence of MCS has steadily increased. The proportion increased from 1.8% in 2000 to 2.8% in 2010, and 3.5% in 2020 [4]. Everyday reliance on fragranced products, cleaning agents, and other harmful household chemicals further degrades indoor air quality, exposing individuals in settings presumed to be safe, such as homes, workplaces, and healthcare settings. Despite the scale of impact, public awareness remains limited, and policymakers require clear evidence of effective measures to improve air quality, reduce harm, and ensure accessibility and equity. Unfortunately, this evidence often fails to address the disproportionate harm to vulnerable populations, such as individuals with MCS whose health and quality of life depend on clean air, leaving them exposed to intolerable conditions [5].

The target audience for the conference reflected this broad societal need: policymakers, standards and curriculum developers, researchers, medical educators and students, human rights advocates, equity-seeking organizations, health professionals, the private sector, people living with MCS, and the general public. The two-day conference was designed with dual objectives. The first day highlighted the biological mechanisms and clinical manifestations of MCS, underscoring the need for improved diagnostic criteria and therapeutic strategies, and addressed gaps in scientific and medical recognition. Day 2 focused on social and legal perspectives, examining public attitudes, the effectiveness of scent-free policies, and the obligations of both the public and private sectors to uphold the rights of persons with MCS. Together, these discussions reinforced the imperative to implement accommodation for the recognized disability of MCS, ensuring accessibility and removing systemic barriers to participation. 

## 2. Conference Sections

### 2.1. Day 1

The day began with an opening address by Dino Zuppa, PhD, Chief Executive Officer of Accessibility Standards Canada (ASC), who outlined the organization’s mandate and its central role in developing national accessibility standards. Under the Accessible Canada Act, ASC is committed to achieving a barrier-free Canada by 2040. Its mission is guided by four key principles: developing accessibility standards, funding research, serving as a centre of expertise, and supporting both regulatory and non-regulatory initiatives. Reflecting this commitment to equity, 90% of ASC’s contributors are members of equity-deserving groups, positioning the organization as a leader in inclusion, diversity, and accessibility.

With ASC’s support, ASEQ-EHAQ has conducted extensive research on indoor air quality-work that was further showcased on Day 2. Findings demonstrate that VOC levels are significantly elevated in workplaces lacking scent-free policies, and that individuals living with MCS face restricted access to the built environment due to both systemic and attitudinal barriers. Inadequate product labelling further complicates consumers’ and institutions’ efforts to make safe, informed choices. Attitudinal barriers, in particular, reflect widespread gaps in public understanding and persistent stigmatization of people living with MCS, including disbelief in the legitimacy of chemical-related health impacts.

The remainder of the day focused on directly addressing these attitudinal barriers and reinforcing the validity and seriousness of MCS.

The keynote speaker of the day, John Molot, MD, a researcher in environmental medicine, presented his paper, “*Multiple Chemical Sensitivity: It’s Time to Catch Up to the Science*,” offering a comprehensive overview of MCS and its biological foundations. A central focus of his presentation was the Transient Receptor Potential (TRP) family of receptors. Drawing on 21 human studies and 16 animal studies, Dr. Molot highlighted evidence showing that two TRP subfamilies-TRPV1 and TRPA1-can become sensitized following repeated exposure to chemicals and pollutants. Such sensitization and over-activation lead to oxidative stress, inflammation, and cellular damage at exposure levels well below conventional thresholds. The ubiquity of TRP channels across multiple body systems, combined with the symptoms triggered through their activation, provides strong support for the biological etiology of MCS.

On a related note, Kenichi Azuma, PhD, and Kentaro Watai, MD, PhD, distinguished researchers from Japan, presented groundbreaking research on the pathophysiology of MCS. Dr. Azuma highlighted findings from functional brain imaging studies demonstrating abnormal neural responses in individuals with MCS following exposure to low-level odours and chemicals [6]. Chemical exposure is encoded differently in people with MCS compared to controls. This differential encoding is amplified by networks and stored differently in memory, as shown in earlier work [7]. Notably, Dr. Azuma noted that olfactory sensitivity itself is not significantly different between affected and unaffected individuals; rather, the distinction lies in how odours are processed further along neural pathways-mechanisms that ultimately drive MCS symptoms.

Following this, Dr. Watai presented his research on the gut–brain–immune axis in MCS, using gut microbiome profiling and genetic analysis [8]. His study identified distinct gut microbiota compositions among individuals with MCS, potentially reflecting differences in immune function and Multiple Chemical Sensitivity. Findings from genome-wide association studies (GWAS) further suggest that genetic variants, particularly those related to immune regulation and metabolic pathways, may contribute to individual susceptibility to MCS.

Together, the presentations underscored that people with MCS exhibit innate biological differences. Speakers emphasized that current research supports the interpretation of MCS as involving identifiable biological pathways and challenged interpretations that frame it primarily as a psychological condition. Both researchers emphasized the urgent need for further investigation to clarify the mechanisms that differentiate individuals who develop MCS from those who do not.

Building on this, Audrey Grant, PhD, a genomics researcher at McGill University, is conducting research aimed precisely at addressing this question. One emerging hypothesis is that genetic differences may increase an individual’s propensity to develop MCS. Dr. Molot presented impaired detoxification [3] as a possible mechanism of MCS. This hypothesis suggests that certain individuals may be less able to metabolize and eliminate chemicals from their bodies. Reduced detoxification capacity could lead to prolonged internal exposure, oxidative stress, sensitization of TRPV1 and TRPA1 receptors, and ultimately the development of MCS. 

Dr. Grant presented a comprehensive review of genetic studies related to MCS, identifying several single-nucleotide polymorphisms (SNPs) associated with heightened chemical sensitivity. Notably, these genetic differences may occur in detoxification enzymes (such as members of the CYP450 family) and/or in cellular receptors involved in chemical sensing (such as TRP channels). She also introduced her ongoing research collaboration with Genome Québec and ASEQ-EHAQ, which aims to identify genetic and epigenetic markers that may predict susceptibility to MCS.

Her presentation emphasized that advancing this line of research could help explain the substantial individual variability in MCS and symptom presentation among people with MCS. Ultimately, this project seeks to develop early screening tools and strengthen the recognition of MCS as a biologically grounded condition.

To present another hypothesis of MCS pathophysiology, Haris Theoharides, MD, PhD introduced Mast Cell Activation Syndrome (MCAS), a condition with notable parallels to MCS [9]. In his presentation, he discussed potential overlap between MCAS and MCS, suggesting that symptoms of MCS may be driven, in part, by inappropriate mast cell activation, particularly in tissues where environmental triggers are first encountered, such as the skin, airways, gastrointestinal tract, and brain [10].

Mast cells release a wide range of inflammatory mediators, including histamine, cytokines, chemokines, and prostaglandins, which can provoke inflammation, oxidative stress, and cell damage, mechanisms similar to those described for TRP channel sensitization [11]. Both TRPV1 and TRPA1 receptors are expressed on mast cells and are involved with their activation and degranulation [12,13,14]. Importantly, mast cells are highly responsive to many of the same environmental triggers implicated in MCS, including pesticides, volatile organic compounds (VOCs), particulate matter, and other pollutants [15]. Their widespread presence across body systems means that chronic or repeated stimulation may contribute to the sensitization patterns and multi-system symptoms reported by individuals with MCS.

This perspective aligns with understanding MCS as a neuro-immune condition shaped by interactions among immune activation, stress pathways, and chemical exposures [16]. Dr. Theoharides emphasized that a comparative analysis of MCS and MCAS may help illuminate shared biological pathways, further supporting the interpretation that MCS reflects physiological changes triggered by chemical exposures rather than psychological causes.

To further strengthen the evidence that MCS symptoms arise from chemical and pollutant exposures, Shahir Masri, PhD, Associate Specialist in Air Pollution Exposure Assessment & Epidemiology at the University of California, presented his research on shared exposure events. Dr. Masri described theToxicant-Induced Loss of Tolerance (TILT) model, a two-phase framework proposed to explain the onset and progression of MCS. According to TILT, the initiation phase occurs when a significant exposure to chemicals or pollutants (such as pesticides or industrial contaminants) disrupts the body’s tolerance mechanisms. Following this sensitization, the triggering phase emerges: even very low-level exposures to unrelated everyday substances (e.g., fragrances, cleaning agents) can provoke disabling, multisystem symptoms.

Dr. Masri reviewed eight real-world exposure events, including industrial spills, Gulf War exposures, and building-related illness cases, in which clusters of individuals subsequently developed MCS-like symptoms [17]. Many affected individuals reported long-lasting chemical intolerance following a single high-level exposure or repeated moderate exposures. Notably, a substantial proportion of affected individuals were reported to experience symptoms consistent with MCS years after the initiating event. The cases reviewed were presented as illustrative examples suggesting that environmental toxicant exposure may contribute to the development of MCS and may help explain sensitization patterns observed in clinical contexts.

Building on this, another potential source of chemical exposure discussed was dental materials. Ottaviano Tapparo, MD, a doctor of medical dentistry, presented his research on the toxic effects of dental fillings and on diagnostic tools that may help identify and monitor oral sources of chemical exposure relevant to MCS. Dr. Tapparo emphasized that the biocompatibility of dental materials is often overlooked in conventional dentistry, yet may play a critical role in worsening or sustaining MCS symptoms. In individuals with MCS, even low-level chemical releases from common dental materials, such as metals, amalgams, and resin-based composites, may provoke systemic reactions due to heightened sensitivity and impaired detoxification mechanisms.

To minimize exposure risks, Dr. Tapparo recommended updating current dental protocols for patients with MCS. He described several preliminary assessment techniques that may help identify problematic materials before dental treatment, including: a chewing-gum stimulation test to detect the release of metals or other substances from existing dental work; blood tests that assess inflammatory markers, immune reactivity, or potential chemical hypersensitivities; and the Maintrac^®^ test, which may provide insight into abnormal immune activity or early indicators of systemic stress associated with environmental exposures. Together, these approaches could support more personalized, precautionary dental care for individuals with MCS.

Representing the Ottawa Environmental Health Clinic, Jennifer Armstrong, MD, presented a physician’s perspective on MCS and shared key insights on appropriate patient care and condition management. Since 1997, Dr. Armstrong has treated more than 3000 patients in a patient-centred, integrative, and chemical-free clinical environment. She emphasized that MCS is a complex, multi-system condition that requires a holistic and individualized approach, one that is often absent in conventional medical settings.

Dr. Armstrong advocated for a paradigm shift in medicine, urging clinicians to move away from standardized pharmaceutical-based approaches and toward personalized treatment plans tailored to each patient’s biology, exposures, and lived experience. She illustrated her points with clinical case studies and long-term trends observed in her practice.

She also provided an overview of the history of environmental medicine, highlighting the work of Theron Randolph, MD, widely regarded as the founder of the field. Using conceptual diagrams, Dr. Armstrong explained how repeated chemical exposures can gradually push the body from adaptive functioning into progressive dysfunction and, ultimately, end-organ impairment. This model, she noted, aligns closely with patterns she has observed in clinical practice and offers a compelling explanation for how chronic illness develops and persists in environmentally sensitive individuals.

Dr. Armstrong argued that effective MCS management should focus on reducing toxic load, supporting natural detoxification pathways, and restoring immune balance, particularly because many individuals with MCS react poorly to conventional pharmaceuticals. Her presentation served both as a clinical roadmap and a call to action, inviting medical professionals, educators, and policymakers to adopt a model of care that listens to patients, respects individual variability, and acknowledges the biological reality of MCS.

The day concluded with a panel discussion featuring MCS researchers, air quality experts, and physicians specializing in environmental medicine. Panellists explored scientific and research barriers, gaps in the built environment, limitations of current standards, the need for education and awareness, and pathways for strategic action. A recurring theme was the absence of a clear medical identifier for MCS, which contributes to widespread misunderstanding across both public and private sectors. As a result, insufficient resources are directed toward studying the condition, and many, including physicians, continue to misinterpret MCS as a psychological issue. This creates a detrimental feedback loop that fuels stigma, undermines the legitimacy of patient experiences, and delays appropriate care.

Panellists emphasized that most healthcare providers, educators, and policymakers remain unaware that MCS is a legitimate health condition. They stressed that MCS must be incorporated into medical and nursing school curricula, with future professionals trained to recognize and appropriately respond to MCS.

Policy reform emerged as a critical priority, particularly the development of universal accessibility standards that account for Multiple Chemical Sensitivity. The panel called for global guidelines and regulatory frameworks that explicitly recognize MCS, providing essential clarity for governments, standard developers, employers, and healthcare systems. Such measures would support improved diagnostic approaches, spur therapeutic research, and further solidify the medical and scientific legitimacy of MCS.

The central theme of Day 1 was the presentation of scientific evidence that speakers argued demonstrates advancing understanding of the biological mechanisms potentially underlying MCS. Across the day, speakers highlighted key scientific domains, including receptor sensitivity, gene-environment interactions, chemical exposure pathways, proposed mechanisms of pathophysiology, and emerging treatment strategies. Speakers repeatedly described MCS as a multi-system, multi-factorial condition, whose diagnosis, management, and treatment must reflect this complexity.

Many presenters advocated for a systems biology approach, integrating microbiome, genetic, metabolic, and environmental exposure data to improve diagnostic precision and therapeutic strategies. Speakers suggested that this integrated perspective reflects the interaction of biological predispositions and environmental triggers, rather than psychological or subjective causes.

One central theme gained universal agreement: the next critical step in advancing the field is the development of an objective diagnostic measure for MCS. At present, diagnosis relies heavily on the Quick Environmental Exposure and Sensitivity Inventory (QEESI), a validated but self-reported questionnaire that quantifies chemical intolerance. While QEESI is a valuable tool, researchers emphasized that biomarkers or physiological tests would greatly enhance diagnostic accuracy, improve scientific acceptance, and guide more effective clinical care.

Across presentations on Day 1, speakers highlighted growing scientific evidence suggesting that biological mechanisms may play a central role in MCS. Discussions emphasized evolving frameworks integrating genetics, environmental health, neuro-immune pathways, and toxicology as areas of active investigation.

### 2.2. Day 2

Representing ASC, Dr. Zuppa opened the second day of the event. He revisited the organization’s role and founding principles, this time emphasizing its collaboration with EHAQ on national indoor air quality testing to inform new housing and built-environment standards. He presented findings from the study evaluating the effectiveness of scent-free workplace policies by comparing environments with and without such measures. Remarkably, workplaces enforcing scent-free policies showed a 70% reduction in volatile organic compound (VOC) concentrations, according to ASC-supported indoor air quality research presented at the conference. In his opening remarks, Dr. Zuppa emphasized that even straightforward, well-implemented policies can yield substantial improvements in indoor air quality.

Dr. Zuppa further noted that scent-free environments offer benefits beyond MCS, improving conditions for individuals with asthma, chronic migraines, and chronic fatigue. Interestingly, spaces that were frequently cleaned or treated sometimes had poorer air quality due to emissions from cleaning products. The presentation underscored the urgency of translating research into standards governing HVAC performance, construction materials, and safe cleaning protocols.

He outlined ASC’s approach to developing actionable outcomes: reviewing existing standards through innovative research, creating technical requirements, and using ASC’s inclusive standard-setting process to embed MCS-related considerations into future policies. Dr. Zuppa reaffirmed ASC’s commitment to positioning Canada as a global leader in standards development.

He also acknowledged the challenges ahead. While Day 1 highlighted attitudinal barriers, Day 2 brought attention to the systemic barriers affecting individuals with MCS. Issues such as scent-free policies, bias in scientific discourse, and the broader recognition of MCS as a disability were central themes explored by both speakers and participants.

Before examining the systemic barriers associated with MCS, the global prevalence and impact of the condition were powerfully highlighted. While MCS is well established as a growing public health concern worldwide [1], statistics alone cannot convey the depth of the lived experience. To bring these realities forward, EHAC-ASEC and ASEQ-EHAQ collaborated with MCS organizations from France, Spain, the United Kingdom, Italy, Germany, Japan, Scotland, and Australia to produce a video illustrating the profound challenges faced by individuals with MCS, the limited resources available, the responsibilities of policymakers, and the collective hope for change.

Across regions, communities reported strikingly similar obstacles: denied diagnoses, a lack of management and treatment options, inadequate housing and shelter, and widespread gaps in medical knowledge. These barriers often lead to cascading consequences, stigma, economic instability, deteriorating health, social isolation, and chronic stress. In Canada, the situation has reached a level where some individuals have been advised to consider Medical Assistance in Dying (MAiD) due to the absence of safe housing and care.

Organizations around the world described systemic patterns of neglect that hinder public recognition of MCS and restrict access to essential services, including healthcare. In response, the collective message from all participating groups was unequivocal: there is an urgent need for strengthened international collaboration, harmonized policy and research efforts, and increased visibility of MCS within disability rights movements and public health planning.

To further examine the situation in Canada, Rohini Peris, President & CEO of ASEQ-EHAQ and EHAC-ASEC, presented findings from the Canadian Community Health Survey (CCHS). According to the 2020 CCHS, approximately 3.5% of Canadians have a diagnosed case of MCS, and the data reveal that diagnoses have more than doubled over the past decade, signalling a rapidly escalating public health issue.

Peris also highlighted several critical sociodemographic patterns. Women were more likely to report symptoms of MCS, yet paradoxically less likely to receive a formal diagnosis. Individuals living in lower-income households face higher levels of exposure to environmental toxins and have fewer resources to mitigate risk or seek medical evaluation. As a result, MCS disproportionately affects marginalized communities and those of lower socioeconomic status.

Peris emphasized the need for fragrance-free healthcare environments, safer and more accessible housing options, and strengthened accessibility legislation that explicitly includes MCS. Although MCS is often invisible to others, its impact on the daily lives of those affected is profound.

The presentation underscored the urgency of closing the recognition gap between the well-documented prevalence of MCS and the lack of institutional response in medical practice, housing policy, and public health systems.

However, as the CEO of ASEQ-EHAQ and EHAC-ASEC, Peris has led multiple national research initiatives generating both qualitative and quantitative evidence to support scent-free policies and strengthen recognition of the MCS community. Two of these projects were presented during the conference.

The first presentation, delivered by researchers Nene Diallo, MSc, and Adrianna Trifunovski, MSc, expanded on Dr. Zuppa’s conclusion that scent-free policies can reduce harmful VOC levels by up to 70%, including significant reductions in acetaldehyde, benzene and formaldehyde, three known carcinogens. This conclusion was the result of a preliminary study comparing indoor air quality in scent-free environments and those without such a policy. Despite the clear benefits of scent-free policies, the study identified substantial gaps in implementation. Many workplaces that claimed to be scent-free lacked training, monitoring, and enforcement mechanisms. These “policy implementation gaps” were compounded by widespread misconceptions, for example, the belief that scented products “clean the air” or that synthetic fragrances are interchangeable with natural scents. The findings revealed that users of fragranced products are generally unaware of their chemical composition and associated health risks.

A second major initiative, ASEQ-EHAQ’s flagship Empowering Communities and Removal of Barriers (ECRoB) project, was highlighted by Susan Yousafzai, MSc. Through focus groups, the study explored the everyday barriers faced by people living with MCS. Participants commonly reported medical neglect, lack of access to essential services, and resistance to providing accommodations. These systemic failures contribute to stress, identity loss, isolation, and avoidance of public spaces. The research further revealed that misinformation circulating through media and advertising fosters the misconception that MCS is psychogenic, reinforcing stigma and disbelief.

Together, these studies call for stronger product transparency, public education campaigns, and integrated research across science, law, and public health. They underscore the urgent need for coordinated action to recognize MCS as a legitimate disability and to ensure safe, inclusive environments for those affected.

To further examine public understanding and the widespread gaps in awareness regarding household chemicals, Caroline Barakat, PhD, an environmental health professor at Ontario Tech University, presented findings from her recent study. She highlighted that fragranced products, cleaning supplies, furniture, and cosmetics commonly contain highly toxic chemical substances. These may include parabens, phthalates, triclosan, lead, bisphenol A, and perchloroethylene, all of which pose significant risks to the brain, endocrine system, and other internal organs.

Through interviews with women, Dr. Barakat assessed knowledge of these hazards. Strikingly, most participants were completely unaware of the risks associated with routine chemical exposures. Their understanding was shaped almost entirely by social media narratives and manufacturer-driven advertising campaigns, sources that frequently distort or downplay toxicity. Notably, once participants became aware of the risks, many adopted avoidance behaviours, reduced their use of harmful products, and subsequently improved indoor air quality, demonstrating that education can meaningfully shift behaviour.

The study also revealed that sociodemographic factors such as income, education level, and ethnic background play a moderating role in risk perception and response. Individuals from minority or lower-income communities were less likely to be aware of chemical hazards and less likely to adopt exposure-reduction strategies. Financial and material constraints may force reliance on inexpensive, and often more heavily scented or hazardous, products, inadvertently increasing vulnerability to chemical sensitivity.

Dr. Barakat underscored the urgent need for effective, evidence-based educational tools that improve public literacy on chemical exposures. Such interventions should be grounded in behavioural change models, clearly convey essential information, and ideally be delivered through interactive digital platforms to maximize accessibility and impact.

While Dr. Barakat underscored the urgent need for public education, Dr. Molot addressed why such education has not been meaningfully initiated within the scientific community itself. His presentation highlighted the pervasive bias within parts of the scientific and medical fields regarding MCS. Despite a growing body of evidence demonstrating that MCS is a legitimate, biologically rooted condition, many scientists continue to dismiss its validity.

Dr. Molot referenced the Semmelweis reflex, the tendency to reject new information that challenges established norms, even when that information is evidence-based. He explained how implicit bias and confirmation bias shape how research findings are interpreted: data consistent with pre-existing beliefs is accepted, while contradictory evidence is minimized or disregarded. The situation is further compounded by manipulation, disinformation, and misinformation, which distort scientific facts and contribute to the ongoing marginalization of people living with MCS.

He also noted the absence of official, evidence-based clinical guidelines for MCS, a gap that reflects broader institutional failures. Dr. Molot highlighted the longstanding inaction of governments despite repeated recommendations from environmental health experts. He cited Ontario’s lack of progress and the 2021 INSPQ report, which he argued overlooked key scientific findings and dismissed as anxiety the gender disparities central to the condition.

He emphasized that local and provincial governments have rarely consulted subject-matter experts with expertise in MCS and have frequently misrepresented the scientific landscape when addressing the condition. Legal systems have similarly been influenced by medical bias, with many individuals unable to secure fair hearings due to the absence of formally recognized expertise.

Dr. Molot concluded by stressing that chemical avoidance remains the only evidence-based intervention for reducing symptoms and improving quality of life for individuals with MCS. Yet neither the scientific community nor political institutions have taken sufficient steps to promote or require chemical- and scent-free environments, despite clear evidence that such measures reduce exposure and eliminate barriers.

To illustrate how MCS is treated in both the courtroom and the workplace, Robert Lattanzio, LLB, and Melissa Pagliaro, MA, presented their perspectives as representatives of the ARCH Disability Law Centre and the Canadian Council on Rehabilitation and Work (CCRW). Pagliaro emphasized that accommodating individuals with disabilities, including those with MCS, is not discretionary but a matter of legal obligation. Lattanzio, Executive Director of ARCH, further noted that ableism continues to shape legal processes, where stigma, misunderstanding, and a lack of awareness about MCS undermine equal access to justice, housing, healthcare, and employment.

Robert Lattanzio described an internal review of 700 legal decisions involving MCS and reported patterns of systemic bias in the adjudication of these cases, including, he said, the dismissal of MCS-specific medical evidence. As Dr. Molot similarly observed, tribunals and courts rarely consult experts in MCS research, and evaluators often question the legitimacy of patient symptoms. Compounding this are procedural barriers: the absence of fragrance-free courtrooms, the lack of chemical-free spaces, and the limited availability of remote or virtual participation options. These gaps effectively restrict access to justice and heighten fear of stigma and disbelief among people with MCS.

From a workplace perspective, CCRW highlighted effective inclusion strategies such as fragrance-free policies, flexible or remote work arrangements, and safer cleaning protocols. Despite common misconceptions about the financial burden, these measures are generally low-cost, straightforward to implement, and have consistently received positive feedback from both employees and employers after adoption.

Lattanzio and Pagliaro advocated for a rights-based, systemic approach that includes legal reform, clearer procedural accommodations for MCS, stronger employee protections, and structural changes to build a more inclusive, equitable society.

Like the first, Day 2 also concluded with a panel discussion, this time bringing together leaders from Canadian disability organizations to address the status of MCS as a disability and outline next steps to support the broader MCS community. Panellists described the profound isolation, exclusion, and daily inaccessibility experienced by individuals with MCS due to pervasive chemical exposures in homes, workplaces, healthcare settings, and public spaces. The absence of systemic recognition remains one of the greatest barriers, and speakers emphasized the urgent need for a coordinated national approach, rather than fragmented or localized initiatives.

From an employment perspective, chemical exposure in workplaces was identified as a major driver of disability-related exclusion. Panellists stressed the need for employer education, inclusive hiring practices, and the adoption of federally supported scent-free and low-emission workplace policies. Environmental health was also framed as an equity issue, with calls for community-led, intersectional strategies that reflect the realities of marginalized populations who face compounded risks.

It was noted that MCS frequently co-occurs with other disabilities, including autism and sensory disorders, and that cross-disability collaboration can amplify shared advocacy efforts and strengthen the push for systemic reform. The panel articulated clear priorities moving forward: establishing federal scent-free and low-emission policies, promoting cross-sector and cross-disability partnerships, expanding public and community-based education, and ensuring that people with MCS are directly involved in designing policies, programs, and standards.

Ultimately, integrating environmental health into Canada’s accessibility framework was presented as essential, not only to address the needs of those living with MCS but to create healthier and more inclusive environments for all Canadians.

The day concluded with a keynote address from Paul-Claude Bérubé, LLB, former CEO of Accessibility Standards Canada and Independent Living Canada, who delivered a powerful closing message. He acknowledged the lack of legislative progress to address MCS and chemical exposures, despite the growing body of scientific evidence documenting their causes, symptoms, and long-term impacts. While science has advanced, he noted, institutional and political action have not kept pace.

Bérubé called for reflection, accountability, and meaningful action from decision-makers. He drew a compelling parallel between chemical exposure today and the historical normalization of smoking. Decades ago, smoking was permitted in workplaces, hospitals, airplanes, and public spaces. Once research exposed its harms, society moved toward strong policies that protected non-smokers and reshaped collective norms. Likewise, he argued, fragranced products and chemical exposures must be treated as preventable hazards, requiring policy intervention to safeguard public health and ensure accessibility.

Day 2 of the conference made clear that MCS is highly prevalent and has profound impacts. There is an urgent need for governments and major institutions to adopt policies that prevent harmful exposures and stop the progression of MCS. Overcoming longstanding biases requires a willingness to examine the extensive research conducted by environmental health experts and the lived experience of affected individuals.

Organizations such as ASEQ-EHAQ, EHAC-ASEC, and counterparts across Canada and internationally are leading the charge for evidence-based policy reform. Speakers throughout the conference described MCS as an emerging international public health concern, and the qualitative perspectives shared in this conference underscore the need for greater public awareness and federal laws mandating scent-free and low-emission environments to ensure accessibility for all disabilities.

## 3. Concluding Remarks

Ultimately, the *Resilience: International Conference on Multiple Chemical Sensitivity* served as a defining first step in a growing global movement. ASEQ-EHAQ and EHAC-ASEC successfully leveraged their expertise and partnerships, spanning governmental agencies, disability organizations, disability law centres, and international MCS groups, to illuminate a rapidly escalating public health crisis. The event was groundbreaking, drawing 870 attendees from around the world, including Canada, the United States, the United Kingdom, France, Australia, Germany, Ireland, Scotland, Israel, Japan, Spain, Italy, Switzerland, and Morocco. The conference sought to catalyze discussion regarding legal reform, healthcare education, and public policy change, while providing participants with a scientific framework to inform ongoing dialogue and advocacy efforts. Speakers throughout the conference emphasized the need for greater recognition of MCS within healthcare policy frameworks, as well as for policy updates to improve accessibility and reduce exposure-related risks.

By the conclusion of the event, attendees had gained a deeper understanding of the causes, symptomatology, and biological mechanisms of MCS, including the role of gene-environment interactions in its onset and progression. Participants also learned about the rapid rise in global prevalence, the lived experiences of individuals with MCS, and the measurable impact of scent-free policies, alongside the systemic barriers that have prevented their widespread adoption. The conference illuminated the profound social, environmental, economic, and political challenges faced by those living with MCS.

Across both days, the rich panel discussions highlighted the urgent next steps required to advance equity, accessibility, and inclusion for all disabilities, particularly for those whose needs have long been overlooked in policy and practice.

The Environmental Health Association of Québec and the Environmental Health Association of Canada organized this event to unite, inform, and inspire hope within the broader MCS community. This conference marks only the beginning. Much more work lies ahead, and far more knowledge will continue to be shared to confront a condition that will continue to grow and harm future generations if silence remains the prevailing response.

## Data Availability

The original contributions presented in this study are included in the article. Further inquiries can be directed to the corresponding author.
